# Benserazide, a dopadecarboxylase inhibitor, suppresses tumor growth by targeting hexokinase 2

**DOI:** 10.1186/s13046-017-0530-4

**Published:** 2017-04-20

**Authors:** Wei Li, Mengzhu Zheng, Shuangping Wu, Suyu Gao, Mei Yang, Zhimei Li, Qiuxia Min, Weiguang Sun, Lixia Chen, Guangya Xiang, Hua Li

**Affiliations:** 1 Hubei Key Laboratory of Natural Medicinal Chemistry and Resource Evaluation, School of Pharmacy, Tongji Medical College, Huazhong University of Science and Technology, Wuhan, 430030 China; 2 School of Traditional Chinese Materia Medica, Wuya College of Innovation, Key Laboratory of Structure-Based Drug Design and Discovery, Ministry of Education, Shenyang Pharmaceutical University, Shenyang, 110016 China; 3 School of Public Health, Wuhan University of Science and Technology, Wuhan, 430081 China

**Keywords:** Benserazide, Colorectal cancer, Drug repurposing, HK2 inhibitor, Structure-based virtual ligand screening

## Abstract

**Background:**

Hexokinase (HK) is the rate-limiting enzyme in the first reaction of glycolysis. And Hexokinase 2 (HK2) is most closely related to malignant tumor which expresses at higher level compared with normal cells. HK2 plays a pivotal role in tumor initiation and maintenance, which provides a new target for cancer therapy.

**Methods:**

Structure-based virtual ligand screening was used in hit identification from ZINC Drug Database. Microscale thermophoresis assay was performed to evaluate the binding affinity. Enzyme inhibition, cytotoxicity, apoptosis, intracellular ATP level, mitochondrial membrane potential (MMP), glucose uptake and lactate production experiments were undertaken in SW480 cells to identify Benz as a HK2 inhibitor. Western blot was used to test protein expression. SW480 cells xenograft mouse models were used for in vivo study. Nano-particles of Benz were prepared to improve the antitumor efficacy and tumor targeting of Benz. HPLC was used to measure the concentration of free Benz in tumor tissues.

**Results:**

Benserazide (Benz), was identified as a selective HK2 inhibitor, could specifically bind to HK2 and significantly inhibit HK2 enzymatic activity in vitro. In addition, Benz reduced glucose uptake, lactate production and intracellular ATP level, and could cause cell apoptosis and an increased loss of MMP as well. In vivo study indicated that intraperitoneal (ip) injection of Benz at 300 and 600 mg/Kg suppressed cancer growth in tumor-bearing mice and no toxicity shown. To further improve the antitumor efficacy and tumor targeting of Benz, nano-particles of Benz was prepared. Liposomal Benz at 100 and 200 mg/Kg performed potent inhibitory effects on tumor-bearing mice, showing reduced dose and better efficacy.

**Conclusions:**

Our study provides a new direction for the development of Benz and its analogues as novel antitumor agents for cancer therapy.

**Electronic supplementary material:**

The online version of this article (doi:10.1186/s13046-017-0530-4) contains supplementary material, which is available to authorized users.

## Background

Colorectal cancer (CRC) is the third most common cancer with 1.2 million new cases and 0.6 million deaths per year worldwide and is frequently diagnosed at advanced stages [1]. In United States, during 2009 to 2013, the incidence rates of colon cancer were 46.9 and 35.6 per 100, 000 for male and female. Moreover, 17.7 and 12.4 per 100, 000 for male and female were death to colon cancer [2]. The high incidence, high fatality rate and poor prognosis of CRC make it a disease that seriously influences people’s health. The efficacy of current therapies including endoscopic removal of precursor lesions and chemotherapeutic treatments are limited for CRC treatment [3]. Therefore, searching for more effective anticancer agents is urgent for CRC therapy. Warburg effect was discovered in 1930s and widespread in solid tumor cells such as colorectal cancer, breast cancer and liver cancer and so on. Warburg effect is one of the most fundamental metabolic alterations during tumor development and progression, of which the over-expressed hexokinase 2 (HK2) plays a crucial role in glycolytic pathway [4–6]. HK2 is the enzyme controlling the first step of glycolysis, and systemic deletion of HK2 can impair the tumor progression in mouse models. Moreover, the expression level of HK2 distinguishes cancer cells from normal cells, which provides exciting opportunities for the development of therapeutic strategies to preferentially kill cancer cells [7–9].

Although HK2 is a novel anti-tumor target, there are few potent HK2 inhibitors identified. Nowadays, Metformin (Met), 2-Deoxyglucose (2-DG) and 3-Bromopyruvate (3-BrPA) are most commonly reported HK2 inhibitors. Met, a widely used anti-diabetic drug, is confirmed that it also exerts anti-tumor effect on several cancers [10–15]. Owing to the weak effect of Met in vitro or vivo, a series of novel Met derivates are synthesized, aiming at improving the antitumor activity [16]. 2-DG is a product-mediated HK2 inhibitor but it does not have significant therapeutic activity at the dose range of 500-2000 mg/Kg in mice as a single agent [17]. As for 3-BrPA, which is a halogenated pyruvate and strong alkylating agent toward the free SH groups of cysteine residues in proteins, it is not surprising that it may affect multiple enzymes and causes unavoidable side-effects. In addition, 3-BrPA is unstable and only exhibits the inhibition of glycolysis at a high concentration [18]. Because of the poor effect and side-effects of these compounds, developing novel potent HK2 inhibitors for treatment of CRC or other cancers is a matter of great urgency. So in our study, structure-based virtual ligand screening method was adopted to screen the FDA-approved drug database and Benserazide (Benz) was identified as an HK2 inhibitor. Benz, a peripheral dopadecarboxylase inhibitor, was often used in combination with levodopa for the treatment of Parkinson’s disease. The definite pharmacokinetics, pharmacodynamics and low toxicity of Benz largely encouraged the developing of Benz or its derivatives as anti-tumor agents.

## Methods

### Reagents

Benz for injection was purchased from Dalian Meilun Biology Technology Co., Ltd (China). The glucose-6-phosphate dehydrogenase (G6P-DH) was purchased from “Roche” (Switzerland). Antibodies against Hexokinase 2, AMPKα and p-AMPKα were obtained from “Cell Signaling Technology” (USA). And β-actin, Bcl-2, Bax and horseradish peroxidase (HRP) conjugated secondary antibody from rabbit were purchased from Wuxi UcallM Biotechnology Co., Ltd (China). Recombinant HK1 protein was obtained from Shanghai yuanye Bio-Technology Co., Ltd (China). All other common chemicals, solvents and reagents were of highest grade available from various commercial sources.

### Preparation of nano-particles of Benz

Firstly, HSPC/Chol (molar ratio 55:45) was putted into CHCl_3_ and dried to a thin film on a rotary evaporator. Then the dried lipid mixture and Benz were rehydrated in phosphate buffer (PBS, pH 4.0) at 60 °C for 1 h in flowing nitrogen. The suspended vesicles were extruded through the 200 nm and 100 nm pore-size polycarbonate membranes at least five times using a Lipex extruder (Canada), and then purified by size exclusion chromatography to remove free Benz.

### Freeze-drying of liposomes

The laboratory freeze drier (Germany) was applied for freeze-drying and detailed process was as follows: (1) freezing at -40 °C for 8 h; (2) primary drying at -40 °C for 48 h; (3) secondary drying at 25 °C for 10 h. And the chamber pressure was maintained at 20 pascals during the drying process. HPLC was used to measure the concentration of the Benz. The mean diameter and polydispersity index (PDI) of liposomes were determined by Nano Brook Zeta PALS (Brookhaven Instruments Corporation, USA), based on the dynamic light-scattering principle technique.

### Expression and purification of HK2 and HK4

Genes encoding HK2 (Genebank: BC021116.1) and HK4 (Genebank: BC001890.1) were cloned into pET-26b vector respectively. The sequence-verified recombinant plasmids were transformed into *E. coli* BL21 (DE3) (Invitrogen) and then cultured in LB medium at 37 °C, induced by 0.4 mM isopropyl-*D*-thiogalactopyranoside (IPTG) at 20 °C for 24 h. Subsequently, bacterial suspension was harvested and lysed by ultrasonification. After high speed centrifugation, the protein was purified with Ni-agarose affinity chromatography, followed by anion exchange chromatography and size exclusion chromatography.

### In vitro enzyme inhibition assay

To assay the inhibitory effects of Benz on HK2, HK1 and HK4, NADH which was generated through a coupled reaction with glucose-6-phosphate dehydrogenase (G6P-DH) was detected at 340 nm. Briefly, 10 *μ*L recombinant HK2 (1 *μ*M), HK4 (1 *μ*M) or HK1 (1 *μ*M) was incubated with 5 *μ*L Benz at 37 °C for 10 min. Then 85 *μ*L assay mix containing 100 mM Tris HCl pH 8.0, 5 mM MgCl_2_, 200 mM Glucose, 0.8 mM ATP, 1 mM NAD^+^, 0.25 Units of G6P-DH, was added. The enzyme inhibition IC_50_ values for Benz were calculated based on the changes in absorbance. No Benz interference with G6P-DH activity was observed.

### Microscale thermophoresis (MST) assay

MST was used to analyze the binding affinity between potential ligands and receptors. The purified HK2 was labeled with the Monolith NT™ Protein Labeling Kit RED (Cat # L001) according to the supplied labeling protocol. Labelled HK2 was kept constant at 20 nM, and all tested samples were 1:1 diluted in a 20 mM HEPES (pH 7.4) and 0.05 (v/v) % Tween-20. After 10 min incubation at room temperature, samples were loaded into Monolith™ standard-treated capillaries. And the thermophoresis was measured at 25 °C after 30 min incubation on a Monolith NT.115 instrument (Germany). Furthermore, laser power was set to 20% or 40% using 30 sec on-time. The LED power was set to 100%. The dissociation constant K_d_ values were fitted by using the NTAnalysis software.

### Molecular docking

Crystal structure of human HK2 (PDB code: 2NZT) was obtained from the Protein Data Bank. The docking was operated by ICM 3.8.2 modeling software on an Intel i7 4960 processor (MolSoft LLC, San Diego, CA). And ligand binding pocket residues were selected by graphical tools in ICM software to create the boundaries of docking search. The glucose was the co-crystallized ligand in this crystal structure for docking, and it was deleted when setting up the receptor. In docking calculation, potential energy maps of the receptor were calculated using default parameters. Compounds were inputted into ICM and an index file was created. Conformational sampling was based on the Monte Carlo procedure30, and finally the lowest-energy and the most favorable orientation of the ligand was selected [19, 20].

### Cell cultures

Human colon cancer cells SW480 (ATCC # CCL-228™) were obtained from the American Type Culture Collection (ATCC, USA). Human colon cancer cells (HCT116, Lovo), human breast cancer cells (MCF-7), human hepatoma carcinoma cells (SMMC-7721), human hepatic cells (LO2) and african green monkey kidney kidney cells (Vero) were purchased from BOSTER (Wuhan, China) and cultured in high Glucose Dulbecco’s modified Eagle’s medium supplemented with 2 mM glutamine, 10% (v/v) fetal bovine serum (FBS), 100 U/mL penicillin and 100 mg/mL streptomycin. Cell cultures were grown and maintained at 37 °C in the humidified tissue culture incubator with 5% CO_2_ using standard tissue procedures.

### Cytotoxicity test

Cancer cells (SW480, Lovo, HCT116, MCF-7, SMMC-7721) and normal cells (LO2, Vero) were incubated with 0-500 *μ*M Benz for 48 h and the cytotoxicity of Benz was measured using a Cell Counting Kit-8 (CCK-8). Met was used as a positive control. All experiments were performed in triplicate.

### Cell proliferation assay

HCT116 and SW480 cells were seeded at a density of 2 × 10^3^ per well into 96-well plates. Cell proliferation was assessed by incubating them with Cell Counting Kit-8 reagents (Dojindo, Shanghai, China) for 24 h,48 h, 72 h, 96 h at 37 °C and measuring the absorbance at 450 nm. The experiment was performed in triplicate.

### HK2 siRNA in SW480 cancer cells

SW480 cells were transfected with either two siRNAs against HK2 or one nontargeting siRNA and cultured in 6-well plates according to the manufacturer’s instructions. The target sequences of oligo siRNAs were as follows: siRNA: 5'- CCAAAGACATCTCAGACATTG -3', nontargeting siRNA (Negative control, NC): 5'- UUCUCCGAACGUGUCACGUTT -3' [21].

### Western blot analysis

The harvested cells were lysed with radioimmunoprecipitation assay (RIPA) buffer (Beyotime, China). Insoluble debris was removed by centrifugation and the concentration of total proteins was determined using BCA Protein Assay Kit (Beyotime, China). Then lysate protein (20-40 *μ*g) was subjected to 10% SDS-PAGE and electrophoretically transferred to polyvinylidene difluoride membranes (PVDF) (Millipore, USA). The membranes were sequentially blocked with 5% non-fat milk and incubated overnight with the following primary antibodies: β-actin (A2228), HK2 (#2867), Bcl-2 (DR0564), Bax (DR0549), AMPKα (#5832), p-AMPKα (#2535). Protein bands were visualized using an enhanced chemiluminescence reagent (ECL Plus) (GE Healthcare, USA) after hybridization with a HRP conjugated secondary antibody. All experiments were performed in triplicate and analyzed by optical density.

### Glucose uptake assay

The Glucose Assay Kit (Rsbio, Shanghai, China) was used to analyse the glucose uptake. After 48 h drug treatment, media was collected and diluted within water. The amount of glucose in the media was then detected. Glucose uptake was determined by subtracting the amount of glucose in each sample from the total amount of glucose in the media (without cells). Met was used as a standard control in this assay.

### Determination of lactate generation

After 48 h drug treatment, media was collected and assayed following the manufacturer’s instructions of Lactic Acid Production Detection kit (KeyGen, Nanjing, China) to measure the generation of lactic acid. The assay was determined using a plate reader (Synergy HT, BioTek, USA) at 530 nm. Met was used as a positive control.

### Measurement of intracellular ATP level

Benz-treated (75, 150, 300 *μ*M for 48 h) and Met-treated SW480 cells were harvested and sonicated in ice three times for 1 min. After centrifugation, supernatants were assayed by ATP assay kit (KeyGen, Nanjing, China) to determine the generation of ATP. The assay was determined using the plate reader (Synergy HT, BioTek, USA) at 636 nm.

### Flow cytometric analysis for apoptosis

SW480 cells were cultured in six-well plates and treated with different concentrations of Benz for 48 h. Then the cells were harvested, washed twice with ice-cold PBS, and mixed in 100 *μ*L of 1× binding buffer (10 mM HEPES/ NaOH, pH 7.4, 140 mM NaCl, 2.5 mM CaCl_2_). After culturing for 15 min at room temperature in Annexin-V/PI double staining liquid (Nanjing KeyGen Biotech. Inc.), the cells were examined by flow cytometry (BD Biosciences, FACSCalibur).

### JC-1 staining to determinate mitochondrial membrane potential

SW480 cells were plated in six-well plates and given various concentrations of Benz treatment for 48 h. After staining with 10 *μ*g/mL JC-1 (JC-1 Mitochondrial Membrane Potential Detection Kit; Beyotime) for 20 min in the incubator (37 °C, 5% CO_2_), cells were rinsed with HBSS twice to remove the nonspecific background staining. Then cells were analyzed using a flow cytometer (BD Biosciences, FACSCalibur), and cells emitting a bright red fluorescence represented the aggregate mitochondria.

### Soft agar colony formation assay

About 0.6% agar was poured into the six-well plate. After solidification, 6 × 10^4^ SW480 cells, which were resuspended in 1 mL of 0.3% agar, were overlayed onto the bottom agar and treated with Benz at different concentrations. Media was changed every three days, and colonies were further observed for 10 days. Colonies were then stained with 0.01% of crystal violet staining solution for 2 h.

### Antitumor efficacy of Benz in xenograft mouse model in vivo

All animal experiments were performed in accordance with the Guide for the Care and Use of Laboratory Animals of Tongji Medical College, Huazhong University of Science and Technology and approved by the Ethics Committee. CB-17/ SCID mice (male, 4 weeks old) were purchased from Beijing HFK Bioscience CO., LTD (Beijing, China). SW480 cells were inoculated subcutaneously (3 × 10^6^ cells) into the left flank of each mouse. Six days later, mice were randomly divided into six groups and were injected i.p. daily for 16 days with one of the following treatments: (1) natural saline group (*n* = 10); (2) 300 mg/Kg Benz (*n* = 10); (3) 600 mg/Kg Benz (*n* = 10); (4) vehicle group (*n* = 10); (5) 100 mg/Kg liposomal Benz-treated group (*n* = 10); (6) 200 mg/Kg liposomal Benz-treated group (*n* = 10). The dose volume was 0.1 mL/10 g body weight, and the weights of mice were recorded every day. Meanwhile, the tumor volumes were measured with vernier calipers and calculated by the following formula: (A × B^2^)/2, where A was length and B was width of the two-dimension tumor. After animals were sacrificed, tumor weights were measured. The inhibition ratio (%) was calculated using the following equation: I% = 100% × [W_tumor (vehicle)_ − W_tumor (treated)_]/W_tumor (vehicle)_

### Detection of free Benz in tumor tissue

After recording the weights of tumor mass, tumor tissues from 600 mg/Kg Benz-treated group and 200 mg/Kg liposomal Benz-treated group were lightly washed and blotted to remove any excess blood. Then tumor tissues were homogenized and extracted with methanol, and 100 *μ*L of the extracts were then subjected to HPLC assay. HPLC analysis was conducted by using a reverse phase column (Restek C18 5 *μ*m, 250 mm × 4.6 mm) with a mobile phase of methanol-water- trifluoroacetic acid (20: 1000: 1). Free Benz was detected by measuring the absorbance at 220 nm.

### TUNEL and H&E staining assay

Apoptosis of tumor tissues was detected by terminal deoxynucleotidyl transferase (TdT)-mediated dUTP-biotin nick end labeling (TUNEL) stain, using an In Situ Cell Death Detection Kit (Roche, Switzerland). The hematoxylin-eosin (H&E) staining was performed to observe the pathological changes in different groups.

## Results

### Identifying the binding of Benz to HK2 by virtual ligand screening

**Fig. 1:**
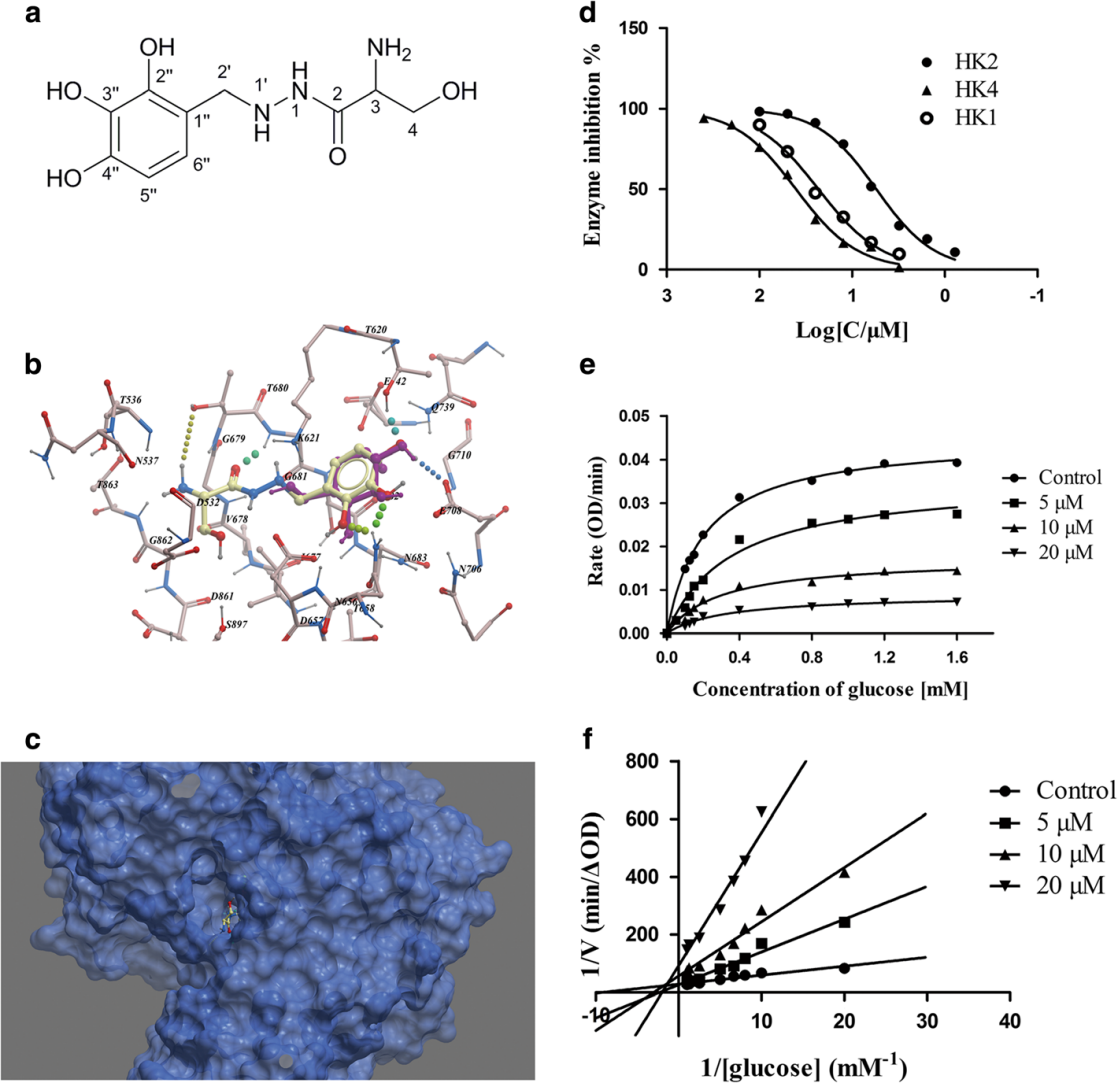
Benz binds to HK2 and inhibits HK2 enzyme activity. **a** Chemical structure of Benz. **b** H-bond interactions between Benz and HK2 residues. Benz and substrate glucose are depicted as ball-and-stick model. **c** Benz occupies the binding site of the substrate glucose **d** Selectivity enzymatic inhibitory efficacy against HK2 versus HK4 and HK1. **e**- **f** The alteration of maximal reaction rate (V_max_) and kinetic constant K_m_. All data presented were the mean ± SEM of three independent experiments

Two thousand nine hundred twenty four approved drugs and nutraceuticals from ZINC Drug Database were screened *in silico*. And eight screening hits with significant ICM scores were selected for further evaluation. Among the eight compounds, Benserazide (Benz) exhibited the strongest enzyme inhibitory effects. As displayed in Table 1, Benz showed both significant ICM scores and high occupancy of the active binding pocket. As shown in Fig. 1b-c, molecule docking result revealed that Benz adopted an extended conformation, in which pyrogallol part of Benz occupied the binding site of the substrate glucose and appeared as a similar conformation. Six hydrogen bonds were predicted between: 2-carbonyl and Gly681, 3-amino and Thr680, 2′′-hydroxyl and Asn656, 3′′-hydroxyl and Asn656, 4′′-hydroxyl and Thr620, 4′′-hydroxyl and Glu708. There were no hydrophobic interactions formed between Benz and HK2, which was consistent with the highly hydrophilic property of the active site of the enzyme.

**Table 1:** Predicted binding free energies and enzyme inhibitory effects

Compound	ICM scores ^a^ (Kcal/mol)	Binding pocket ^b^	Enzyme IC_50_ (*μ*M)
Benserazide	-35.3	4/5, Y	5.52 ± 0.17
D-Fructose	-37.6	1/3, Y	n.i ^C^
Meglumine	-34.4	1/3, Y	>1000
D-Glucosamine	-39.2	1/3, Y	>1000
Vitamin C	-37.7	1/3, Y	>1000
Chlorhexidine	-33.6	N	n.i
Sulfasalazine	-29.7	N	>1000
Pamidronate	-28.1	1/10, Y	n.i

^a^Docking scores of compounds with HK2

^b^Y, yes; Docking into the binding pocket. N, no; Not docking into the binding pocket. Fractions represent the occupancy

^c^n.i, means no enzyme inhibition

### Selective inhibitory effects of Benz on HK2, HK1and HK4 enzymatic activity

To explore the inhibitory activity of Benz on HK2 enzyme in vitro, recombinant human HK2 protein was expressed and the enzymatic activity was evaluated by detecting the increase of NADH absorbance at 340 nm [22]. Benz showed inhibitory effects against HK2, HK1 and HK4 with IC_50_ values of 5.52 ± 0.17 *μ*M, 25.13 ± 0.24 *μ*M and 40.53 ± 2.94 *μ*M, respectively (Fig. 1d). As displayed in Fig. 1d, Benz exhibited 8-fold selectivity against HK2 versus HK4 and about 5-fold selectivity against HK2 versus HK1, which indicated the reasonable selectivity and significant inhibitory activities. As shown in Fig. 1f, the kinetic constants (K_m_) values of glucose were increased at a concentration-dependent manner, which suggested a competitive inhibition of Benz to HK2. Meanwhile, the maximal reaction rate (V_max_) values of glucose were decreased in the presence or absence of Benz treatment (Fig. 1e and Table 2), which indicated the characteristics of noncompetitive inhibitory effects. A potential mechanism of inhibition was supposed that Benz bonded to the active site through a conformation change. Taken together, the above results proved that Benz could significantly inhibited HK2 with a combined mechanism of both competitive and noncompetitive binding.

**Table 2:** Effect of Benz treatment of HK2 on kinetic parameters^a^

Benz (*μ*M)	K_m_ (mM)	V_max_ (OD/min)
0	0.21	0.046
5	0.32	0.037
10	0.52	0.029
20	0.65	0.018

^a^K_m_ and V_max_ values were obtained from Lineweaver-Burk plots. The values were the means of results of three experiments

### Specific binding of Benz with HK2

**Fig. 2:**
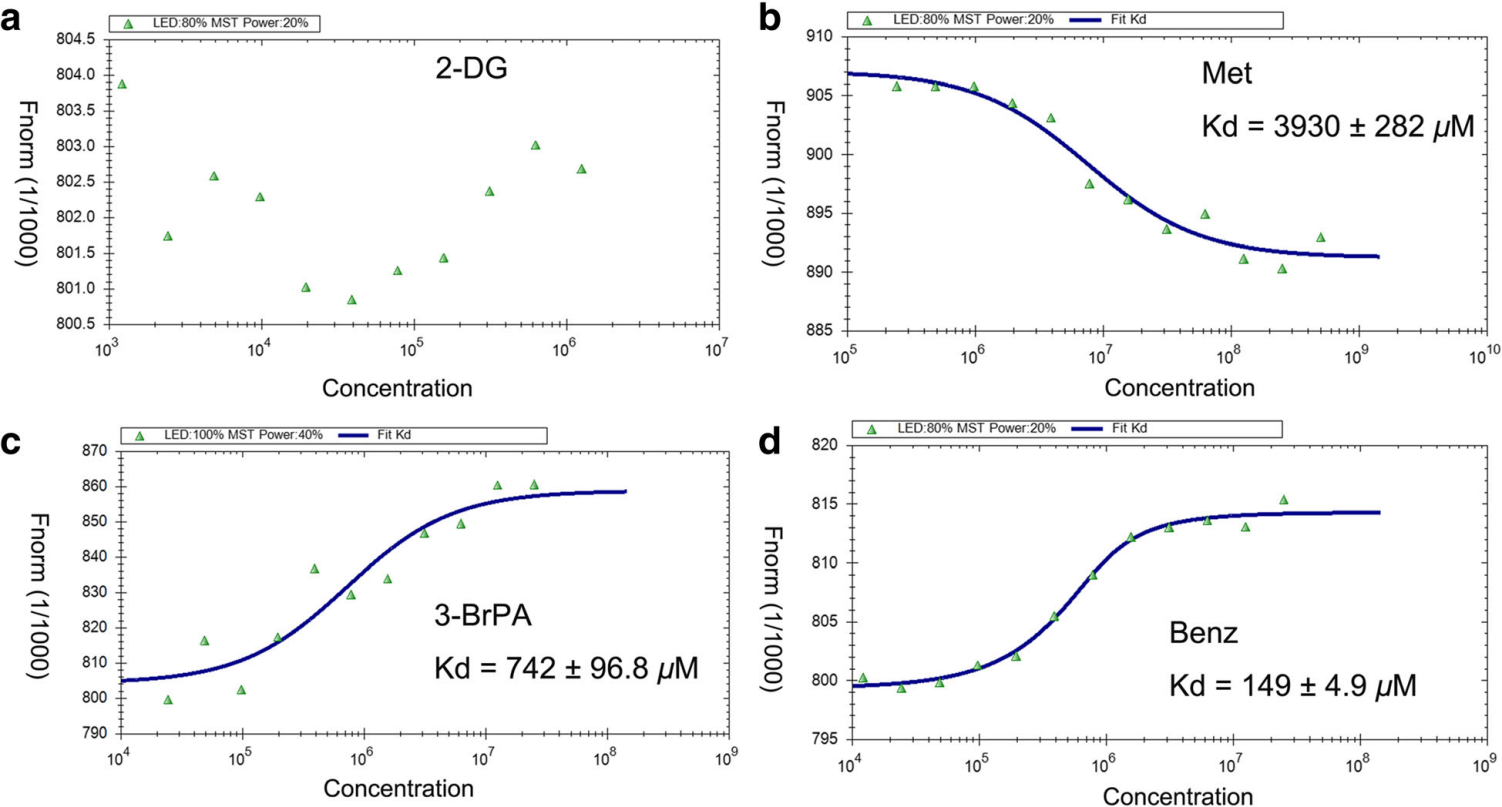
MST assay to measure the binding between compounds and HK2. **a** No binding affinity between 2-DG and HK2 was detected. K_d_ values 3930 ± 282 **b**, 742 ± 96.8 **c**, 149 ± 4.9 **d** *μ*M for Met, 3-BrPA and Benz, respectively. K_d_ values were automatic calculated by the curve fitting, and presented as mean ± SEM

The microscale thermophoresis method (MST) technique had previously been used to assess protein-protein, small organic molecule-protein, nucleic acids-protein and antibody-protein interactions [23–25]. To verify the results of virtual ligand screening, MST was applied to assay the binding affinity between compounds and HK2. Compared with three other reported HK2 inhibitors, Benz displayed the lowest equilibrium dissociation constant (K_d_) of 149 ± 4.95 *μ*M (Fig. 2a-d), which meant the strongest binding affinity.

### Benz inhibits cell proliferation and blocks the anchorage-independent cell growth

**Fig. 3:**
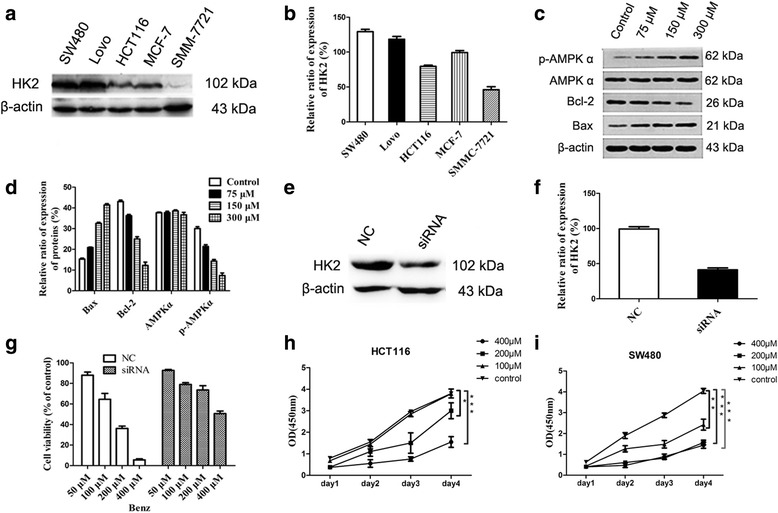
Western blot assay and siRNA knocking down HK2 expression for CCK-8 assay. **a** HK2 expressions in various cancer cells. **b** Quantitative analysis of HK2 expression levels in cancer cells. **c** The expression of glycolysis-related proteins and apoptosis-associated factors: AMPKα, p-AMPKα, Bcl-2 and Bax. **d** Quantitative analysis of expression levels of AMPKα, p-AMPKα, Bcl-2 and Bax. **e** Western blot conforming the suppression of HK2 by siRNA. **f** Quantitative analysis of HK2 expression levels. **g** Knocking down HK2 attenuates the sensitivity of SW480 cells to Benz treatment. All data were shown as means ± SEM for three independent experiments. NC, Negative control. **h**-**i** Effect of Benz on the proliferation of HCT116 and SW480 cells. CCK8 assay was used to detect the cancer cell proliferation after treatment with different concentrations of the Benz (0, 100, 200, 400 *μ*M) for 24, 48, 72 and 96 h. *P < 0.05, **P < 0.01, ***P < 0.001

Before conducting cell experiments, western blot assay was employed to detect the expression level of HK2 in cancer cells. Among the five cancer cells, human colorectal cancer SW480 showed the highest HK2 expression level (Fig. 3a-b). After exposure to Benz and Met treatment for 48 h, the IC_50_ values of cytotoxicity were calculated. As revealed in Table 3, compared with Met, Benz showed higher cytotoxicity to SW480 cells and without notable cytotoxicity to normal cells. Therefore, SW480 cells were selected to evaluate the in vitro or in vivo antitumor activity of Benz in the following experiments. A cell-counting kit-8 (CCK-8) assay was used to detect the cell proliferation after cells had been incubated with different concentrations of Benz (0, 100, 200, 400 *μ*M) for 24, 48, 72 and 96 h. As presented in Fig. 3h and i, CCK-8 assay revealed that Benz markedly inhibited proliferation of both HCT116 and SW480 cells.

**Table 3:** IC_50_ values of Benz and Metformin against various cell lines^a^

Cell lines	IC_50_ of Benz (*μ*M)	IC_50_ of Metformin ^a^ (*μ*M)
SW480	143.0 ± 7.0	>1000
Lovo	151.0 ± 4.5	>1000
HCT116	181.4 ± 11.5	>1000
MCF-7	186.0 ± 12.5	>1000
SMMC-7721	210.4 ± 7.9	>1000
LO2	>500	>1000
Vero	>500	>1000

^a^ Metformin was used as positive control. Data were presented as mean ± SEM for three independent experiments

We further examined the effect of Benz on anchorage-independent growth through colony formation assays. SW480 cells formed spheres on soft agar with saline treatment while 100 *μ*M Benz treatment dramatically reduced the ability to form colonies (Additional file 1: Figure S1), which indicated the Benz-mediated anti-proliferation effect. In short, Benz displayed cytotoxicity and blockage of anchorage-independent cell growth via its HK2 inhibitory effects.

### Knocking down HK2 attenuates the cytotoxicity of Benz

HK2 siRNA was conducted to validate the hypothesis that the cytotoxicity of Benz was mediated directly by targeting HK2. As shown in Fig. 3e-f, HK2 siRNA significantly suppressed the expression of HK2 protein in SW480 cancer cells compared with that of negative control. Benz exhibited cytotoxicity to cells transfected with negative control siRNA but had fewer effects on that of HK2 siRNA transfected cells (Fig. 3g). These results implied that HK2 was a direct target for Benz to exhibit its effects.

### Benz inhibits glycolysis in aerobic glycolytic colorectal cancer cells

**Fig. 4:**
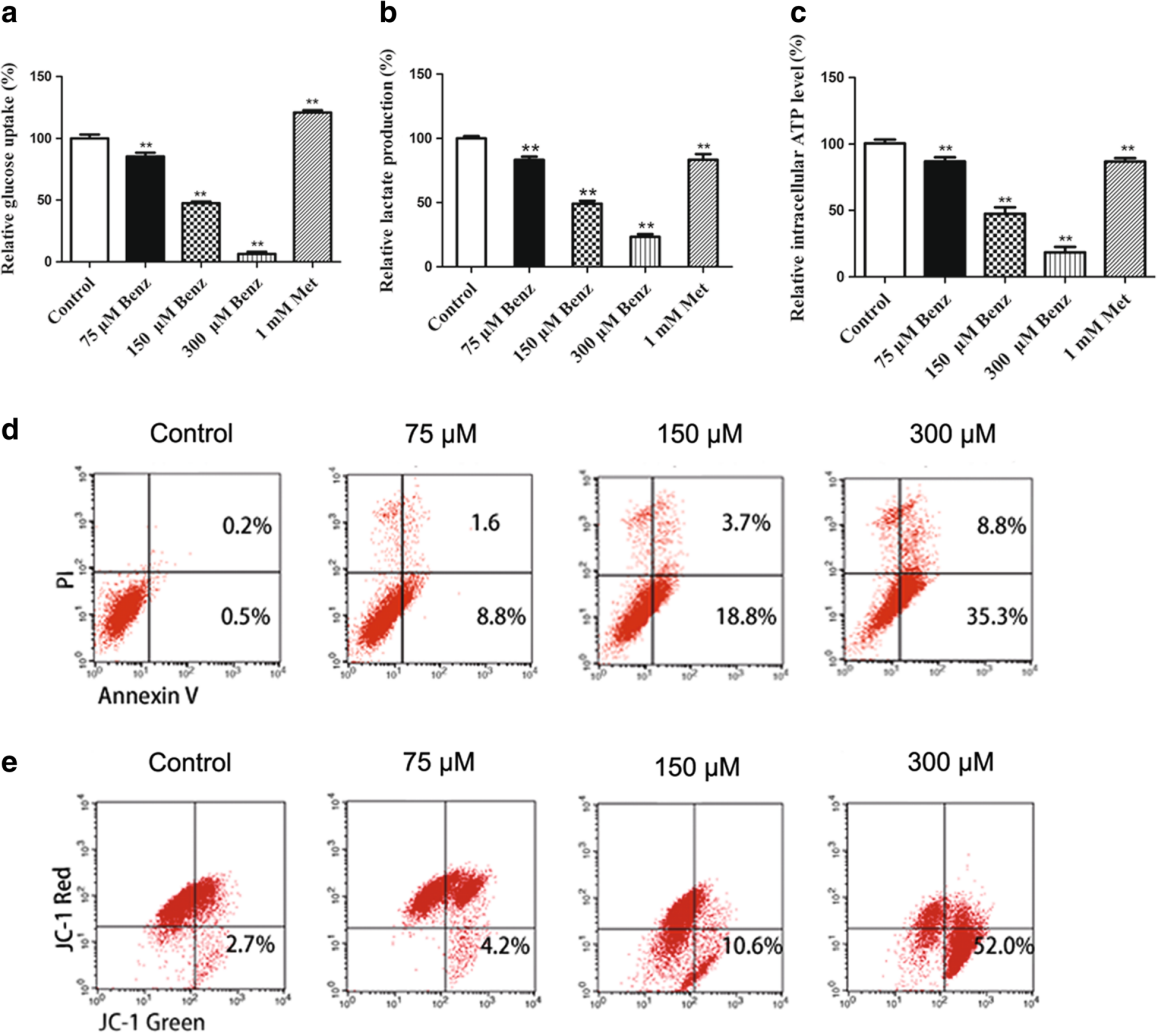
Benz inhibits glycolysis, induces apoptosis and MMP loss in SW480 cells in vitro. **a**-**c** Measurement of glucose uptake, lactate and intracellular ATP level. Metformin is used as positive control. **d** Cell apoptotic death analyzed by Annexin V/PI staining. **e** A significantly loss of MMP in SW480. All data were presented as mean ± SEM for three independent experiments. **P < 0.01 compared with the vehicle control

As shown in Fig. 4a, the glucose uptake in Benz treated SW480 cells was significantly lower than that in untreated or Met treated positive control. In addition, Benz remarkably decreased the concentration of lactate in cell culture supernatant compared to that in untreated control or Met treated positive control (**P < 0.01) (Fig. 4b). As HK2 was a crucial enzyme involved in glycolysis, inhibiting HK2 would reduce the ATP level in cancer cells (**P < 0.01), then triggered activation of the cellular energy regulator AMP-activated protein kinase (AMPK) [26]. Treated with 75, 150 and 300 *μ*M Benz, the intracellular ATP level was reduced while the phosphorylation of AMPKα was increased (Fig. 3c-d and Fig. 4c), which was in agreement with previous studies. Above all, these results suggested that Benz had glycolysis inhibitory effect on cancer cells by targeting HK2.

### Benz induces cell apoptosis

We also explored the mechanisms of Benz-induced growth inhibition by flow cytometry. Annexin-V/PI staining revealed that Benz at 75, 150 and 300 *μ*M induced 10.4%, 22.5% and 44.1% apoptotic death compared with 0.7% in SW480 cells (Fig. 4d). At the same time, the apoptosis-inducing effect of Benz was also confirmed by the altered expression of anti-apoptotic protein Bcl-2 and pro-apoptotic protein Bax (Fig. 3c-d). The potential mechanism was supposed that treatment with Benz could cause energetic stress and then activate AMPKα, p53 and p27, which promoted cell death [27].

### Benz increases the loss of mitochondrial membrane potential

HK2 was tightly bound to mitochondrial and played an important role in maintaining the steady state of mitochondrial membrane potential (MMP) [28]. In vitro enzyme inhibition assay showed that Benz had considerable inhibitory effect. In order to investigate whether this inhibitory effect would cause a loss of MMP, we used JC-1 staining for further verification. And cancer cells treated with Benz at 75, 150 and 300 *μ*M showed an increase of MMP by 4.2%, 10.5% and 52.0%, respectively, compared to that of 2.7% in untreated cells (Fig. 4e). In short, Benz could significantly increase the loss of MMP and its presumed cause was HK2 inhibiting.

### Benz suppresses tumor growth in the CRC xenograft model

**Fig. 5:**
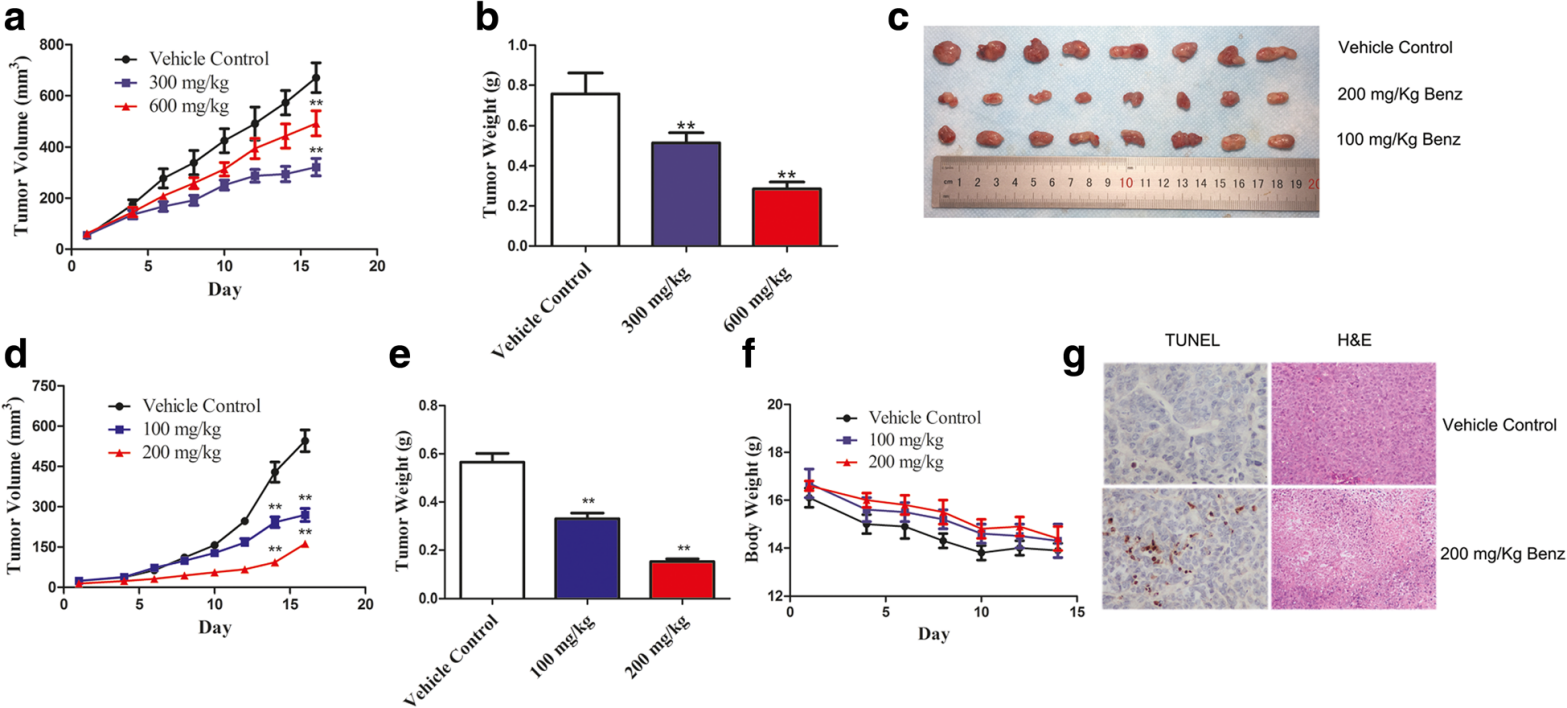
Benz suppresses tumor development in a CRC xenograft model. **a**-**b** Benz impeded tumor development and decreased the size and weight of tumors. **c** Liposomal Benz suppressed tumor development. **d** Tumor volume monitoring after graft. **e** Tumor weight of each group. **f** Measurement of body weight. **g** TUNEL and H&E staining assay of tumor biopsies (100 ×). The differences among groups were compared by analysis of variance (ANOVA). All data were presented as mean ± SEM. **P < 0.01 compared with the vehicle control

To evaluate the effect of Benz in vivo, three million SW480 cells were injected subcutaneously at the left flank of each C.B17/SCID mouse to develop xenograft models. The results showed that Benz hampered tumor growth in mice in comparison to the vehicle treated cohort (Fig. 5a). As shown in Fig. 5b, mice treated with Benz at 300 and 600 mg/Kg per day significantly decreased the size and weight of tumors by 30.1% and 60.3%, respectively, when compared with the vehicle treated cohort (**P < 0.01). After 16 days of Benz treatment, some mice in the control group began sick due to tumor burdens. So, mice were euthanized and tumors were excised for weight measurements and histology analyses. Nano-particles of Ben were prepared to further improve the antitumor efficacy and tumor targeting of Benz. The particle size, Zeta potential and PDI values of Benz liposomes were 150.8 ± 2.5 nm, -0.32 ± 0.06 mV and 0.116 ± 0.054, respectively. The mean entrapment efficiency of Benz was about 20%.

As shown in Fig. 5c-e, mice treated with liposomal Benz at 100 and 200 mg/Kg per day significantly decreased the size and weight of tumors by 41.1% and 73.2%, respectively, when compared with the vehicle treated control (**P < 0.01). To demonstrate the tumor targeting of Benz liposomal, HPLC assay was performed to detect the concentration of free Bnez in tumor tissues. The average concentration of free Benz in Benz-treated group (600 mg/Kg) was 5.59 micrograms per milligram in tumor tissue (*μ*g/mg), which was lower than that of 6.55 *μ*g/mg in liposomal Benz-treated group (200 mg/Kg) (*P* < 0.05). The results showed that even the dose of free Benz was three times of that of liposomal Benz, however, the concentration of the drug in tumor tissues was less. The administration of Benz nano-particles was favorable for drug enrichment in tumor tissues. In addition, no remarkable loss of body weight and change of histomorphology were observed in treated or vehicle-treated mice, which indicated that Benz would be a safe and effective antitumor agent (Fig. 5f and Additional file 1: Figure S2).

### Benz induces tumor apoptosis and pathological changes in vivo

TUNEL assay and H&E staining were applied to further confirm the tumor-suppression effect of Benz in vivo (Fig. 5g). Consistent with the CRC xenograft model animal experiment described above, our results showed that Benz induced tumor apoptosis and pathological changes compared to the vehicle control.

## Discussion

Benz, an FDA-approved decarboxylase inhibitor for adjuvant treatment of Parkinson’s disease, herein was reported to suppress tumor growth by inhibiting HK2. Previous studies revealed that shutting down glucose flux at the earliest step in glucose metabolism by targeting HK2 could be an ideal strategy for cancer therapy [29]. The highly dependency of tumor cells proliferation on accelerated glucose metabolism made them more vulnerable and sensitive to the disturbance of glucose metabolism. Among enzymes involved in glucose metabolism, HK2 played a prominent role in glycolysis. Given its key position in glycolysis catalytic chain and selective overexpression in cancer cells, HK2 provided an attractive target for antitumor drug discovery. However, few HK2 small-molecule inhibitors were discovered due to the extreme polarity of the active site of HK2 and the complexity of its protein functions [30]. Therefore, finding new HK2-targeting candidates is urgent for cancer treatment.

Compared to the ever-increasing failure rates, high cost, and limited efficacy of traditional drug screening approaches, drug repurposing via the analysis of FDA-approved drug was an effective method to identify therapeutic opportunities in cancer and other human diseases [31, 32]. For instance, Sorafenib, a well-known FDA-approved antitumor drug, was identified to be a 5-HT_2A_ inhibitor. Consequently, this compound, as well as other Sorafenib like analogues, had been proposed as potential 5-HT inhibitors [33]. Structure-based virtual ligand screening is a computational method that docks small molecules into the structures of macromolecular targets and scores their potential complementarity to binding sites [34]. Along with great advances in both computational algorithms and computer processing power, this approach is widely used in hit identification and lead optimization. Thus, the combination of structure-based virtual ligand screening and drug repositioning represents an efficient approach to accelerate drug discovery. Because of the verified bioavailability and safety evaluation of approved drugs, the obtained hits have higher probability to enter clinical trials than a new chemical entity.

Benz was identified as an HK2 inhibitor. Besides targeting the decarboxylase, Benz showed inhibitory effect against Coxsackievirus B3 3C protease [35]. Recent studies reported that the pretreatment of Benz enhanced the base-excision DNA repair of oxidative DNA damage in the presence of mutant breast cancer susceptibility gene 1 (BRCA1) [36]. However, the potential mechanisms of these observations remained an area of investigation. In our study, molecule docking result showed that the pyrogallol moiety of Benz occupied the glucose-binding pocket through H-bond interactions and adopted a similar conformation of the substrate glucose. Moreover, in vitro enzymatic inhibition assay and the MST binding assay further conformed the specific targeting of Benz to HK2.

HK1 is highly expressed in normal tissues of the human body, and its distribution is wider than that of HK2 (only expressed in cancer cells). In our in vitro studies, 5.52 *μ*M of Benz can inhibit half of HK2, but it required much more drugs to reach the same extent of inhibition for HK1 and HK4. These results suggested the reasonable selectivity and significant inhibitory activities of Benz. That is to say, when effective concentration of Benz was reached, it was still not enough to significantly decrease the activity of HK1 and HK4, so Benz should not have potentially cytotoxic effects on normal tissues. Furthermore, HK2 siRNA experiments in our study validated the cytotoxicity of Benz was mediated directly by targeting HK2.

As far as we know, Benz had a better binding affinity and efficacy than other known HK2 inhibitors, representing the most potent HK2 inhibitor so far. Furthermore, HK2 located on the outer membrane of mitochondria and contributed to the evasion of apoptosis and the stability of MMP. Consistent with reported studies [5], our data suggested that Benz had the ability to cause an increased loss of MMP and thus induced apoptosis in SW480 cells.

To further improve the antitumor efficacy and tumor targeting of Benz, nano-particles of Benz was prepared. And our results performed that liposomal Benz at the dose of 100 and 200 mg/Kg exhibited potent inhibitory effect on tumor-bearing mice, showing reduced dose and better efficacy. The results showed that the administration dose of free Benz was three times of that of liposomal Benz, but the drug concentration in tumor tissues was much less. If the liposomal NPs only improved the transport efficiency of free Benz, the distribution of Benz in normal tissues should be equal to that of in tumor tissues. However, this may not be true because the higher concentration of the drug was unable to achieve in tumor tissues when the administration dose was only 1/3 of free Benz. So these results suggested that nano-particles of Benz not only further improve the antitumor efficacy but also enhance tumor targeting of Benz.

Benz, as an existing drug with low toxicities, its favorable drug absorption, distribution, metabolism, excretion and toxicity (ADMET) properties made it easy for Benz to expand indication as the antitumor agent. In combination with other anticancer drugs, Benz would reduce the dose of chemotherapy drugs and thus alleviate toxicities and adverse effects. The identification of Benz as a HK2 inhibitor will pave the way for the development of Benz analogues as novel antitumor agents.

## Conclusions

In conclusion, our data indicate that Benz is a good lead compound for designing novel HK2 inhibitors. We are currently conducting *in silico* lead optimization and following synthesis to generate more candidates for in vitro and in vivo evaluation.

## Additional file

Additional file 1: Figure S1. Benz blocks the anchorage-independent cell growth. Images of colony formation of SW480 on soft agar. **Figure S2**. H&E staining assay. Representative photomicrographies (100 ×) of the lung, heart, spleen, kidney and liver of animals from the control group and liposomal Benz-treated group. Hematoxylin-eosin staining was used for visualization and no obvious toxicity in mice was observed. (ZIP 4727 kb)

## Abbreviation

2-DG: 2-Deoxyglucose
3-BrPA: 3-Bromopyruvate
ADMET: Absorption, distribution, metabolism, excretion and toxicity
Benz: Benserazide
BRCA1: Breast cancer susceptibility gene 1
CCK-8: Cell Counting Kit-8
CRC: Colorectal cancer
G6P-DH: Glucose-6-phosphate dehydrogenase
H&E: Hematoxylin-eosin
HK: Hexokinase
HK2: Hexokinase 2
Ip: Intraperitoneal
Met: Metformin
MMP: Mitochondrial membrane potential
MST: Microscale thermophoresis
NC: Negative control
PDI: Polydispersity index
PVDF: Polyvinylidene difluoride membranes
TUNEL: Terminal deoxynucleotidyl transferase (TdT)-mediated dUTP-biotin nick end labeling
