# Supplementary figures and images for: Benserazide, a dopadecarboxylase inhibitor, suppresses tumor growth by targeting hexokinase 2
Source: J Exp Clin Cancer Res. 2017 Apr 20;36:58. doi: 10.1186/s13046-017-0530-4 (PMC5399312; doi:10.1186/s13046-017-0530-4)

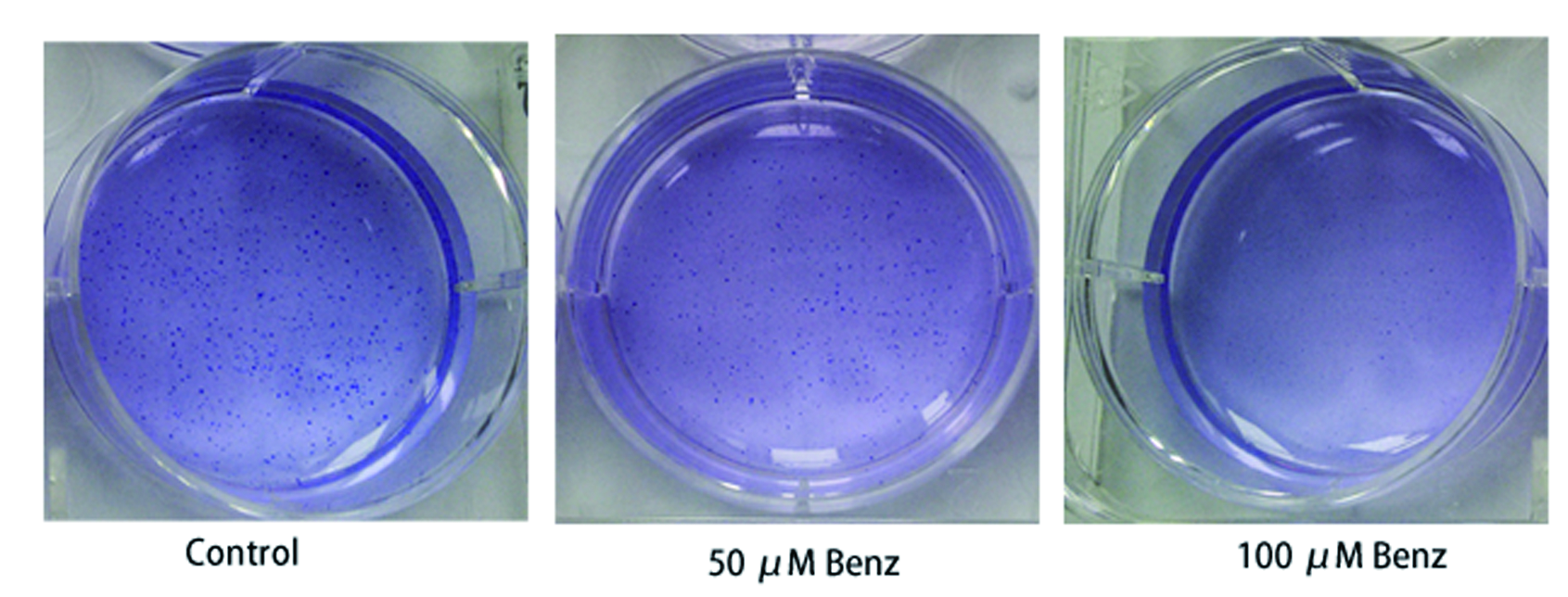

Supplement: Additional file 1: Figure S1. — Benz blocks the anchorage-independent cell growth. Images of colony formation of SW480 on soft agar. Figure S2. H&E staining assay. Representative photomicrographies (100 ×) of the lung, heart, spleen, kidney and liver of animals from the control group and liposomal Benz-treated group. Hematoxylin-eosin staining was used for visualization and no obvious toxicity in mice was observed. (ZIP 4727 kb) [file 13046_2017_530_MOESM1_ESM.zip › Fig S1.tif]

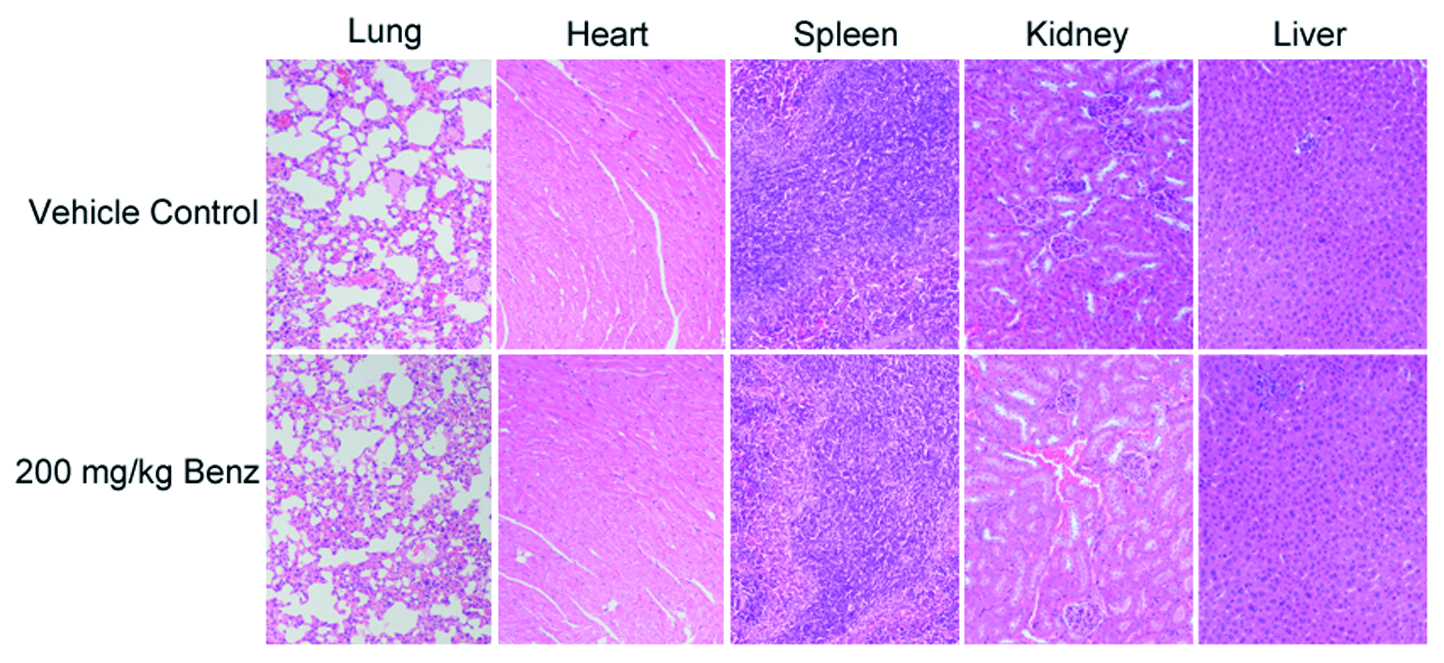

Supplement: Additional file 1: Figure S1. — Benz blocks the anchorage-independent cell growth. Images of colony formation of SW480 on soft agar. Figure S2. H&E staining assay. Representative photomicrographies (100 ×) of the lung, heart, spleen, kidney and liver of animals from the control group and liposomal Benz-treated group. Hematoxylin-eosin staining was used for visualization and no obvious toxicity in mice was observed. (ZIP 4727 kb) [file 13046_2017_530_MOESM1_ESM.zip › Fig S2.tif]
